# Increased Risk of Metabolic Syndrome in Patients with Vitiligo

**DOI:** 10.4274/balkanmedj.2016.1005

**Published:** 2017-05-15

**Authors:** Hatice Ataş, Müzeyyen Gönül

**Affiliations:** 1 Department of Dermatology, University of Health Sciences, Dışkapı Yıldırım Beyazıt Training and Research Hospital, Ankara, Turkey

**Keywords:** metabolic syndrome, vitiligo, screening

## Abstract

**Background:**

Inflammatory and immune processes can be triggered in vitiligo due to a decreased number of melanocytes and their anti-inflammatory effects. Because of the systemic nature of vitiligo, metabolic abnormalities such as insulin resistance and lipid profile disturbances as well as skin involvement may be observed in vitiligo.

**Aims:**

To investigate the association between metabolic syndrome and vitiligo.

**Study Design:**

Case-control study.

**Methods:**

The demographic, clinical and laboratory features in the subjects were compared according to presence of vitiligo and metabolic syndrome [patients (n=63) vs. gender-age matched controls (n=65) and metabolic syndrome positive (n=38) vs. negative (n=90)]. A logistic regression analysis was also used.

**Results:**

We identified metabolic syndrome in 24 (38.1%) subjects with vitiligo and 14 (21.5%) subjects without vitiligo (p=0.04). Active vitiligo, segmental vitiligo, an increased duration of vitiligo and an increased percentage in the affected body surface area were determined to be independent predictors of metabolic syndrome [activity of vitiligo: p=0.012, OR (95% CI)=64.4 (2.5-1672); type of vitiligo: p=0.007, OR (95% CI)=215.1 (4.3-10725.8); duration of vitiligo: p=0.03, OR (95% CI)=1.4 (1.1-2.0); percentage of affected body surface area: p=0.07, OR (95% CI)=1.2 (0.98-1.5)].

**Conclusion:**

The risk of developing metabolic syndrome is increased in patients with vitiligo. The poor clinical features of vitiligo, such as active, extended and segmental vitiligo with an increased duration of time, are independent predictors for developing metabolic syndrome.

Vitiligo is an acquired, progressive, depigmenting disorder which can be divided into non-segmental and segmental classes (1). Segmental vitiligo is characterised by its early onset and poor response to conventional therapies for vitiligo (2). The pathogenesis of vitiligo is largely unknown, but autoimmunity and oxidative stress are two important mechanisms which are responsible for its aetiopathogenesis (3). It is believed that oxidative stress is one of the major reasons for the development of metabolic syndrome (MetS), and can be related to the pathogenesis of certain diseases like vitiligo and psoriasis (4,5,6). Recently, melanocytes have been identified in adipose tissue (7), and it is believed that these melanocytes have anti-inflammatory effects and reduce reactive oxygen species (7). Interestingly, decreases in the number of melanocytes and melanogenesis in the adipose tissue have been reported in vitiligo patients, and it has been suggested that metabolic disorders may develop in these patients (8). However, studies investigating the relationship between vitiligo and MetS are rare in the literature. Based on the above-mentioned information, the aim of this study was to investigate the association between MetS and vitiligo.

## MATERIALS AND METHODS

### Subjects

This was a single centre, case-control study. One-hundred and twenty-eight participants were separated into a patient group (subjects with vitiligo) and a control group (subjects without vitiligo). We selected 63 patients with vitiligo (33 females, 30 males; mean age of 40.1±11.8 years old), and 65 age- and gender-matched controls (34 females, 31 males; mean age of 40.3±10.3 years old) (Table 1). These were admitted to the outpatient clinic of dermatology in order to undergo medical examination. The age- and gender-matched controls were selected from patients admitted to the clinic for minimal dermatological problems, such as nevus and tinea pedis, in order to avoid bias to the study results. The demographic, clinical and laboratory features of the subjects were also compared according to the presence of MetS [MetS positive (n=38) vs. negative (n=90)]. This study received ethics committee approval, and all of the participants gave permission for this research before we began. Some of the inclusion criteria were: depigmentation greater than 10%, older than 18 years, and no systemic or local therapy 3 months before the beginning of the study.

### Affected body surface area

We considered the percentage of the affected body surface area (BSA) as an extension of the disease.

### Disease activity

Stable vitiligo was defined as no change in the lesions detected within 2 months before beginning the study. Active vitiligo was defined as the detection of a new lesion or the enlargement of a previous lesion within 2 months before the study began.

### Type of vitiligo

Vitiligo is divided into 3 types and several subtypes according to the Bordeaux Vitiligo Global Issues Consensus Conference (1):

1. Non-segmental: acrofacial, mucosal (more than one mucosal site), generalised, universal, mixed (associated with segmental vitiligo) and rare variants.

2. Segmental: uni, bi or pluri-segmental.

3. Undetermined/unclassified vitiligo: focal or mucosal (one site in isolation).

### Chemicals

Venous samples were taken from the subjects after 12 hours of fasting, and tested for the glycaemic index, high-density lipoprotein (HDL) and triglycerides using the spectrophotometric method (Siemens Advia-2400; Healthcare Diagnostics Inc., Tarrytown, USA).

### Metabolic syndrome criteria

The MetS criteria were defined by the current (2005) National Cholesterol Education Program Adult Treatment Panel III (ATP III) guidelines, updated by the American Heart Association/National Heart, Lung and Blood Institute (NHLBI) (9). The following criteria were used: waist circumference ≥102 cm for males and ≥88 cm for females, triglycerides ≥150 mg/dL or on treatment, HDL <40 mg/dL for males and <50 mg/dL for females or on treatment, blood pressure ≥130/85 mmHg or on treatment, and glycaemic index ≥100 mg/dL or on treatment. The measuring tape was located on the top of the right iliac crest and placed horizontally around the abdomen without compression to the skin. It was kept parallel to the floor at the end of expiration before reading the measurement. The presence of any three of the five traits in the patient defined MetS.

### Statistical analysis

The Statistical Package for Social Sciences version 15 (SPSS Inc.; Chicago, IL, USA) was used, and the mean ± standard deviation, median and range were calculated for the descriptive data. The Kolmogorov-Smirnov and Shapiro-Wilk’s tests were used for the normal distribution. The chi-squared test or Fisher’s exact test and the Mann-Whitney U test or Student’s t test were used for the comparisons of the groups (patients vs. controls and MetS positive vs. MetS negative) according to normality. The effects of the age, gender, presence of vitiligo, type, duration, percentage of affected BSA and activity of vitiligo on MetS were investigated with univariate and multivariate logistic regression analyses to determine the independent predictors of MetS. Results with p<0.05 showed statistical significance.

## RESULTS

The clinical and laboratory results of this research are presented in Table 1. The mean disease duration and percentage of affected BSA were 9.5±8.1 (1-40) years old and 20.9±11.8% (10-80), respectively. MetS was diagnosed in 24 (38.1%) subjects with vitiligo and 14 (21.5%) subjects without vitiligo. There were no significant differences in the age, gender or other demographic or clinical characteristics, except for MetS, between the vitiligo and control groups (p=0.04) (Table 1). The presence of vitiligo (p=0.04), treatment for hyperglycaemia (p=0.001), age, duration of vitiligo, percentage of affected BSA, systolic and diastolic tension, waist circumference, fasting glycaemic index, triglycerides, HDL and treatment for hypertension (p<0.0001 for each) [except for gender (p=0.41)] were significantly different between the MetS positive and negative groups (Table 1). Active vitiligo, segmental vitiligo, increased duration of vitiligo and increased percentage of the affected BSA were determined as independent predictors for MetS [activity of vitiligo: p=0.012, OR (95% CI)=64.4 (2.5-1672); type of vitiligo: p=0.007, OR (95% CI)=215.1 (4.3-10725.8); duration of vitiligo: p=0.03, OR (95% CI)=1.4 (1.1-2.0); percentage of affected BSA: p=0.07, OR (95% CI)=1.2 (0.98-1.5)] (Table 2).

## DISCUSSION

The pathogenesis of vitiligo is largely unknown; however, changes in the cytokine profiles, autoimmunity and genetic factors can contribute to the start of vitiligo (10,11,12,13). Melanocyte abnormality and destruction, melanin-concentrating hormone (MCH) MCH receptor autoantibodies, the overexpression of MCH, a high level of homocysteine, an increase in catecholamine, free oxygen radicals, cytomegalovirus and stress may be related to the pathogenesis of vitiligo (14,15,16,17,18,19). MetS (insulin resistance syndrome or syndrome X) generally includes abdominal obesity, hyperglycaemia, dyslipidaemia and hypertension (20), and several definitions of MetS have been described by different organisations. However, the NCEP ATP III has provided the most widely used definition (9). Vitiligo is not only restricted to the skin, but is also a systemic disease (5); therefore, there may be metabolic disturbances in cases of vitiligo. Decreased levels of HDL cholesterol and increased triglyceride concentrations have been found in girls with vitiligo when compared to the controls (21). In addition, several studies have reported an increased incidence of vitiligo in diabetic patients (22).

Overall, studies investigating the relationship between vitiligo and MetS in the literature are rare. Insulin resistance with a homeostasis model assessment (HOMA-IR) in vitiligo was evaluated by Karadag et al. (23); however, they did not compare subjects according to the presence of MetS. Higher insulin, C-peptide, HOMA-IR, low-density lipoprotein/HDL, systolic blood pressure and lower HDL levels in vitiligo were reported in their study due to increased cytokines and autoimmunity to melanocytes.

Autoimmunity and oxidative stress in patients with vitiligo can trigger certain systemic manifestations due to inflammatory and immunological pathogeneses, as well as skin involvement. It is believed that an oxidative imbalance is responsible for the development of both MetS and vitiligo (4,5). Melanin in the adipose tissue has anti-inflammatory and antioxidant effects (7). A decreased number of melanocytes as well as decreased melanogenesis in the adipose tissue might reduce the anti-inflammatory effects of the melanocytes, and cause an increased production of the free oxygen radicals in vitiligo, which is responsible for MetS. In addition, other mechanisms may contribute to the development of MetS in patients with vitiligo, such as insulin resistance, lipid profile disturbances and other metabolic disorders, due to the increased inﬂammatory cytokines and autoimmune reactions of the melanocytes. An increased level of homocysteine, which is a tyrosinase inhibitor, may also be a contributing factor to the development of MetS in vitiligo patients (18). These very complicated mechanisms involving many systems may have affected the results of this study, since every system associated with MetS affected by vitiligo may not be affected at the same time. Each system might be affected differently over time.

There is a similar pathological mechanism between vitiligo and MetS (4,5). Overall, the prevalence of MetS is 22% (24), but this has been increasing. MetS research from Turkey has reported the prevalence to be 35%, which is higher in women than in men (25). The lower rate of MetS in the controls can be explained by the lower number in our study than in the cohort studies in the previous literature. Nevertheless, the control rate (21.5%) was compatible with the previous general prevalence. When we analysed the presence of MetS according to the NCEP ATP III, our study revealed that the presence of MetS was higher in the cases of vitiligo (38.1%) than in the controls (21.5%) (p=0.04). Despite the lack of significance between the two groups for the parameters of MetS, the fasting glycaemic index, triglycerides, HDL and systolic tension were higher than in the controls, with the exception of the diastolic tension, treatment for hyperglycaemia and hypertension, and waist circumference. According to our study, the presence of vitiligo was a 2.2 times greater risk factor for MetS in the univariate analysis (OR=2.2, p=0.042). However, it was not found to be an effective independent factor according to the multivariate model. Some of the clinical features of vitiligo, such as active, extended and segmental vitiligo with longer duration times, were more effective on MetS than stable, local, non-segmental and newly diagnosed vitiligo.

An age-dependent increase has been observed for MetS (24). Correspondingly, we also found age to be a possible risk factor (OR=1.1, p<0.0001). However, an increase in MetS due to age can be detected in the general population and in other diseases; therefore, it was not detected as an independent factor in the patients with vitiligo. According to our study, the duration of the vitiligo was more effective than the age.

The prevalence of MetS is higher in women than in men (25). In subjects with MetS, there were more women than men (57.9% vs. 42.1%) in our study, and female predominance was detected in both the control group (57.1% vs. 42.9%) and the patient group (58.3% vs. 41.7%). However, gender was not found to be a risk factor (p=0.41). These results showed that the presence of vitiligo and its poor clinical features, such as active, extended and segmental types of vitiligo with increased duration times, were the most effective parameters on MetS.

Page et al. (7) suggested that the α-melanocyte stimulating hormone, which is an agonist of melanin production, could be used to prevent MetS. HMG-CoA reductase inhibitors (simvastatin) reduce cardiovascular mortality and morbidity, and are used to improve lipid disorders (26). Noel et al. (27) reported an improvement in vitiligo during simvastatin use, due to its immunomodulation effects. The use of antioxidant prophylaxis may be effective for the prevention and treatment of diseases such as vitiligo (28), because the control of the immune system and balance of oxidative/antioxidative pathways for disease remission are important. Enhancing initiatives, such as an increase in melanin production and antioxidative activity, may contribute to decreasing MetS in vitiligo. Therefore, further comparative studies are needed.

In conclusion, this study was conducted to assess the prevalence of MetS in patients with vitiligo, as the comparison and evaluation of MetS with its predictive factors in vitiligo have not been studied before. However, a HOMA-IR evaluation may provide additional contributions and comparisons. Our study shows that the risk for the development of MetS is increased in patients with vitiligo. In addition, our findings suggest that active, extended, segmental vitiligo with an increased duration time is associated with an increased risk for the development of MetS. Screening and the close follow-up of vitiligo patients with poor clinical features, as in active, extended, segmental vitiligo with an increased duration, for the early diagnosis and treatment of MetS, can reduce the morbidity and mortality. This was a preliminary study of MetS in vitiligo patients; therefore, there is a need for more comprehensive studies with larger series and more participants to evaluate MetS in patients with vitiligo.

## Figures and Tables

**Table 1:**
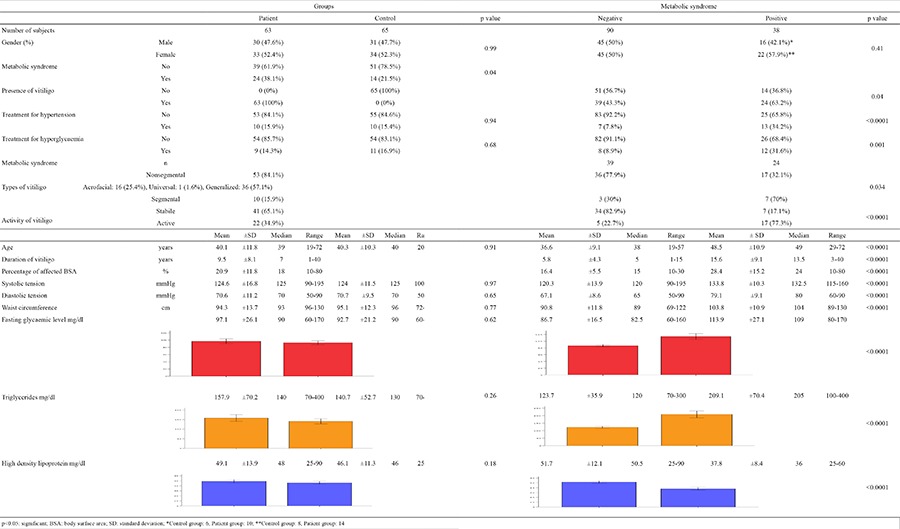
Demographic, clinical and laboratory features according to the presence of vitiligo and metabolic syndrome

**Table 2:**
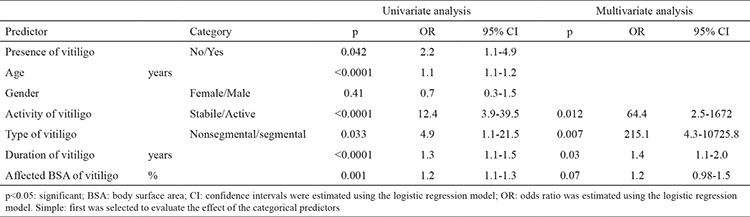
Effects of age, gender, presence, duration, activity, type and extension of vitiligo on metabolic syndrome in the multivariate analysis
